# Alternative Splicing and Protein Diversity: Plants Versus Animals

**DOI:** 10.3389/fpls.2019.00708

**Published:** 2019-06-12

**Authors:** Saurabh Chaudhary, Waqas Khokhar, Ibtissam Jabre, Anireddy S. N. Reddy, Lee J. Byrne, Cornelia M. Wilson, Naeem H. Syed

**Affiliations:** ^1^School of Human and Life Sciences, Canterbury Christ Church University, Canterbury, United Kingdom; ^2^Department of Biology and Program in Cell and Molecular Biology, Colorado State University, Fort Collins, CO, United States

**Keywords:** alternative splicing, co-transcriptional splicing, protein diversity, intron retention, NMD, splicing memory, epigenetic modifications

## Abstract

Plants, unlike animals, exhibit a very high degree of plasticity in their growth and development and employ diverse strategies to cope with the variations during diurnal cycles and stressful conditions. Plants and animals, despite their remarkable morphological and physiological differences, share many basic cellular processes and regulatory mechanisms. Alternative splicing (AS) is one such gene regulatory mechanism that modulates gene expression in multiple ways. It is now well established that AS is prevalent in all multicellular eukaryotes including plants and humans. Emerging evidence indicates that in plants, as in animals, transcription and splicing are coupled. Here, we reviewed recent evidence in support of co-transcriptional splicing in plants and highlighted similarities and differences between plants and humans. An unsettled question in the field of AS is the extent to which splice isoforms contribute to protein diversity. To take a critical look at this question, we presented a comprehensive summary of the current status of research in this area in both plants and humans, discussed limitations with the currently used approaches and suggested improvements to current methods and alternative approaches. We end with a discussion on the potential role of epigenetic modifications and chromatin state in splicing memory in plants primed with stresses.

## Introduction

Plants have evolved various developmental and physiological strategies to control daily activities that respond to variable and extreme environmental conditions (Gratani, 2014; Becklin et al., 2016). To maximize efficiency under diverse conditions, the crosstalk between multiple layers of gene regulation including co-transcriptional, post-transcriptional, and post-translational regulation is crucial for plants (Reddy et al., 2013; Guerra et al., 2015; Skelly et al., 2016). Alternative splicing (AS) is one such mechanism, which is widespread in plants and humans, generates two or more mRNAs from the same precursor-mRNA (pre-mRNA) and is thought to significantly contribute toward protein diversity (Nilsen and Graveley, 2010; Syed et al., 2012; Reddy et al., 2013). The basic mechanism of AS in higher eukaryotes is similar, however, some differences in gene architecture, splicing and transcription machinery between plants and animals suggest plant-specific regulation of AS (Kornblihtt et al., 2013; Irimia and Roy, 2014; Wang et al., 2014).

**FIGURE 1:**
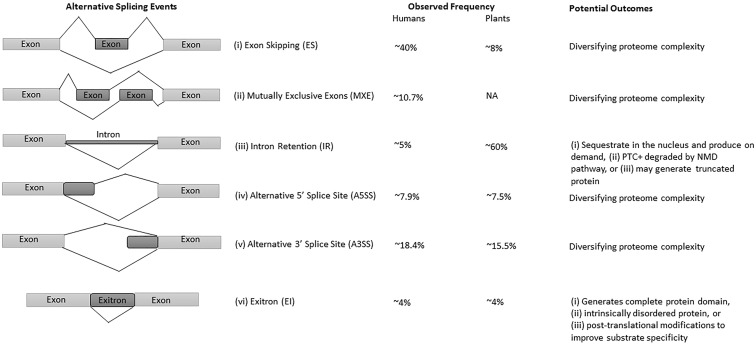
Major types of AS events, their frequency, and potential outcomes in humans and plants. (i) exon skipping (ES) or cassette exon, in which single or multiple exons are spliced out or retained; (ii) mutually exclusive exons (MXE), in which only one of the two exons is retained; (iii) intron retention (IR), where an intron remains in the mature transcript; (iv, v) alternative donor/acceptor site or 5′/3′ splice junction is used to alter the boundary of exons, and (vi) exitrons are a variety of IR with some feature of exons. Constitutive and alternatively spliced exons are represented as light and dark gray blocks, respectively. The observed frequencies represented here are approximate values, and may differ in different species, tissues and conditions. The presented data on AS events frequency are from Reddy et al. (2013), Marquez et al. (2015).

The advances in next-generation sequencing (NGS) technology and omics approaches in plants have revealed that up to 70% of multi-exon genes undergo AS (Filichkin et al., 2010; Lu et al., 2010; Marquez et al., 2012; Shen et al., 2014; Thatcher et al., 2014; Chamala et al., 2015; Zhang et al., 2017). Among all AS events, intron retention (IR) is the predominant mode of AS in plants (Filichkin et al., 2010; Kalyna et al., 2012; Drechsel et al., 2013), whereas exon-skipping (ES) is the major type in humans (Figure 1) (Sammeth et al., 2008; Wang et al., 2008). Interestingly, IR generates mostly non-sense mRNAs harboring premature terminal codons (PTC+) and are either degraded by the non-sense-mediated mRNA decay (NMD) pathway, or escape NMD to produce truncated proteins, thereby regulating the function and abundance of their full-length counterparts (Filichkin and Mockler, 2012; Kalyna et al., 2012; Drechsel et al., 2013; Filichkin S.A. et al., 2015). The NMD pathway is a post-transcriptional mRNA quality control mechanism which acts to degrade PTC+ mRNAs. Some studies suggest alternative roles for transcripts with IR, which are either sequestered in the nucleus and released on demand (Filichkin S.A. et al., 2015; Filichkin S. et al., 2015) or function as protein-coding introns known as exitrons (Figure 1), a new class of retained introns with some features of exons (Marquez et al., 2015; Staiger and Simpson, 2015).

Plants modulate their gene expression patterns via AS coupled to NMD during different developmental stages, abiotic and/or biotic stresses and the circadian clock function (James et al., 2012; Kalyna et al., 2012; Drechsel et al., 2013; Kwon et al., 2014; Filichkin S.A. et al., 2015; Sureshkumar et al., 2016). Stressful conditions control not only the ratios but the timing of both sense and non-sense AS transcripts (Filichkin S.A. et al., 2015; Filichkin et al., 2018). However, it is unclear how environmental signals modulate splicing ratios and timing to help plants acclimate to such stresses in the short and long term. Furthermore, it is largely unknown to what extent AS transcripts are recruited for translation to be functionally significant at the proteomic level in plants.

Alternative splicing regulates essential functions in humans such as autophagy, apoptosis, protein localization, enzymatic activities and interaction with ligands, transcription factors activity and mRNA abundance, etc. (Kelemen et al., 2013; Paronetto et al., 2016; Gallego-Paez et al., 2017). Hence, it is not surprising that any aberrant or dysregulation in AS can cause several human diseases including cancer, neurological disorders, heart, and skeletal muscle abnormalities, and multiple genetic disorders (Matlin et al., 2005; Poulos et al., 2011; Kelemen et al., 2013; Sveen et al., 2016). Recent transcriptome (RNA-Seq), translatome (ribosomal foot-printing), and proteome data have shown a significant contribution of AS toward protein diversity in humans (Weatheritt et al., 2016; Liu et al., 2017). On the other hand, some proteomic studies suggest that AS may not significantly contribute to protein diversity and only single dominant isoforms are represented at the protein level for most of the protein-coding genes (Ezkurdia et al., 2015; Tress et al., 2017a). Apparently, these contradictions stem from the lower depth and limitations of mass spectrometry (MS) techniques to detect changes in protein domains as a result of AS (Wang et al., 2018; Chaudhary et al., 2019). In this review, basic differences in the mechanism of AS and its contribution toward protein diversity in plants and humans are discussed. We also discuss some emerging aspects of IR, NMD pathway, chromatin structure, and splicing memory in plants.

## Coupling of Transcription and Splicing in Plants and Humans

Plant spliceosome machinery is not well characterized due to the unavailability of *in vitro* systems. However, in a recent study, an attempt has been made to develop an *in vitro* pre-mRNA splicing assay using plant nuclear extracts, and it may help to delineate and characterize components of the plant spliceosome machinery (Albaqami and Reddy, 2018). Sequence similarity based analyses suggest conserved regulation of AS in higher eukaryotes. Briefly, splicing is carried out by the spliceosome, which consists of five small nuclear ribonucleoprotein particles (snRNPs) designated as U1, U2, U4, U5, and U6 and additional spliceosome-associated non-snRNP proteins (Will and Lührmann, 2011; Matera and Wang, 2014; Wang et al., 2014). The *cis*-acting elements present on pre-mRNA include 5′ splice sites (5′SS), 3′ splice sites (3′SS), polypyrimidine tracts (PPT) and branch point sequences, which are recognized by the *trans*-acting factors such as splicing factors (SFs) mainly SR proteins and hnRNPs. The *trans*-acting SFs and *cis*-regulatory elements guide and modulate the spliceosome to recognize differential splice sites present on pre-mRNA (Koncz et al., 2012; Reddy et al., 2013; Chen and Moore, 2015). The details on the assembly of the spliceosome and regulation of AS has been reviewed extensively and readers are referred to excellent articles on this topic (Will and Lührmann, 2011; Reddy et al., 2013; Chen and Moore, 2015).

**FIGURE 2:**
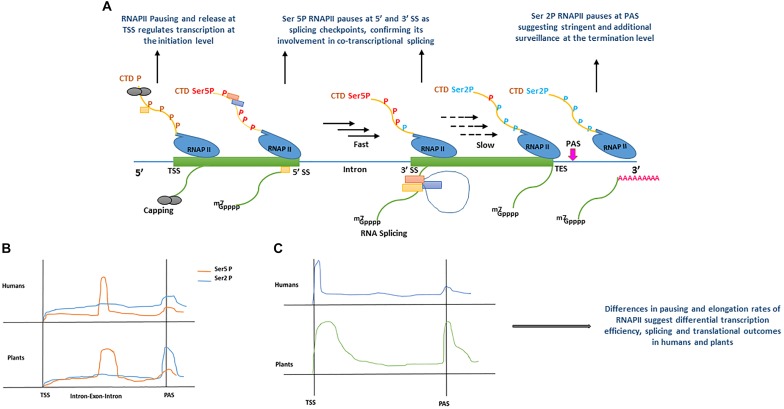
**(A)** Model displaying the role of RNA polymerase II (RNAPII) C-terminal domain (CTD) phosphorylation during co-transcriptional splicing regulation in human (Nojima et al., 2015), and plants (Zhu et al., 2018). During transcription initiation, the serine residues of RNAPII heptad repeat (yellow line) remain un-phosphorylated (brown ‘P’) around transcription start site (TSS) allowing core spliceosome recruitment (yellow rectangle) and capping (gray circles). During the elongation stage, serine 5 residues of RNAPII heptad repeat (red Ser5P) are phosphorylated around the 5′ splice sites (5′ SS) allowing the recruitment of additional components of the spliceosome machinery (orange and blue rectangles) and enhance RNAPII speed (black arrows). RNAPII elongation slows down (black dotted arrows) promotionally with the decrease of Ser5 phosphorylation toward the 3′ splice site (3′SS). Toward the transcription end site (TES), phosphorylation of serine 2 residues increase significantly resulting in RNAPII pausing before mRNA release (green line). m7GPPP and pink ‘repeated A’ represent 5′ cap and poly A tail, respectively. **(B)** Comparison of RNAPII CTD serine 2 and 5 residues phosphorylation levels accumulation between human (Nojima et al., 2015) and plants (Zhu et al., 2018). In human and plants, both serine 5 and serine 2 phosphorylation show significant increase after the transcription start site (TSS), only Ser 5P displays a sharp peak at exon–intron boundaries. For instance, a sharp peak of Ser2 P is only shown at polyadenylation site (PAS) in plants, whereas it remains less prominent in humans. **(C)** Comparison of RNAPII accumulation between humans and plants based on GRO-Seq experiments (Hetzel et al., 2016). In humans and plants, RNAPII occupancy is lower during the elongation stage and marginally increases around PAS. In contrast, plants show a broad peak after TSS, as compared with humans, and a more pronounced increase at PAS, suggesting a surveillance mechanism before a transcript is released. All Graphs are modified from published data to depict peaks.

Recent evidence from metazoans indicates that the process of splicing is largely co-transcriptional (Shukla and Oberdoerffer, 2012; Brugiolo et al., 2013; Merkhofer et al., 2014). Extensive studies in animals and emerging data in plants show that the splicing process for the majority of genes is predominantly co-transcriptional in nature (Figure 2) (Nojima et al., 2015, 2018; Wang et al., 2016; Wong et al., 2017; Zhu et al., 2018; Jabre et al., 2019). The co-transcriptional behavior of splicing means that the chromatin environment such as methylation status, histone modifications, nucleosome occupancy and RNA Polymerase II (RNAPII) processivity has a strong influence on splicing outcomes (Listerman et al., 2006; Khodor et al., 2011; Pajoro et al., 2017; Jabre et al., 2019).

Interestingly, long non-coding RNAs (lncRNAs) can also influence the splicing dynamics of their target genes either directly and/or after processing into short interfering or micro RNAs (Romero-Barrios et al., 2018). Non-coding RNAs can affect AS via modulating chromatin structure (Luco et al., 2011; Romero-Barrios et al., 2018), splicing factor recruitment and altering the phosphorylation status of spliceosomal proteins (Misteli et al., 1998; Romero-Barrios et al., 2018). Circular RNAs which are generated by the so-called non-canonical “backsplicing” of pre-mRNAs are known to regulate AS in animals and examples from plants are beginning to emerge as well. CircRNAs could make DNA:RNA hybrids with the genomic DNA to generate the so-called R-loop. Indeed, a circRNA derived from exon 6 of the *SEPALLATA3* (*SEP3*) gene forms an R-loop via direct interaction with the *SEP3* locus (Conn et al., 2017). The R-loop formation around exon 6 of the *SEP3* gene results in skipping of this exon and affects petal and stamen number in Arabidopsis (Conn et al., 2017).

Plant promoters are largely devoid of nucleosomes, as a result of lower GC content (high AT enrichment) as compared with humans (Narang et al., 2005; Yang et al., 2007; Hetzel et al., 2016). Therefore, the dynamics of transcription initiation are fundamentally different between humans and plants (Hetzel et al., 2016). Depending upon the chromatin context in animals and plants, RNAPII is recruited at a promoter to form the pre-initiation complex (PIC), however, its processivity is inherently dependent on the chromatin structure along gene bodies and influences RNA-processing during transcription (Guo and Price, 2013; Grasser and Grasser, 2018; Jabre et al., 2019). Techniques such as native elongation transcript sequencing (NET-Seq) (Churchman and Weissman, 2011) in mammals (mNET-Seq) (Nojima et al., 2015) and plants (pNET-Seq) (Zhu et al., 2018) and global run-on sequencing (GRO-seq) (Hetzel et al., 2016), have revealed some important aspects of RNAPII elongation and structural features during transcription and RNA-processing, in humans and plants, respectively. The carboxyl-terminal domain (CTD) of the largest subunit of RNAPII contains a heptad repeat “Tyr1-Ser2-Pro3-Thr4-Ser5-Pro6-Ser7.” The Ser2 and Ser5 of this heptad repeat undergoes phosphorylation and plays a key role in the coordination of transcription and other RNA processing activities (Harlen and Churchman, 2017). In mNET-Seq, phosphorylation-specific antibodies were used to study immunoprecipitated RNAPII transcripts in humans (Nojima et al., 2015, 2018). The comparative analysis of un-phosphorylated (unph) or low-phosphorylated and phosphorylated CTD of RNAPII revealed the accumulation of different forms at differential positions on protein-coding genes. For instance, the RNAPII unph-CTD shows a peak at the transcription start site (TSS), whereas RNAPII Ser5P CTD accumulates at the 5′SS of exon–intron boundaries and its density reduces as the RNAPII elongation proceeds downstream toward the 3′ end of the intron (Figure 2A) (Nojima et al., 2015, 2018). Similarly, RNAPII Ser2P CTD spreads over gene bodies (GB) and shows accumulation at the transcription end site (TES) (Figure 2A) (Nojima et al., 2015, 2018). Moreover, genes that undergo co-transcriptional splicing, such as *TARS* in humans, show a major peak of RNAPII Ser5P CTD at 5′SS, suggesting pausing at the exon to allow time for the spliceosome to catalyze the first splicing reaction (Nojima et al., 2015). Similar to humans, the dynamics of RNAPII in plants is also established during transcription (Erhard et al., 2015; Hetzel et al., 2016; Zhu et al., 2018). As shown in the proposed model of co-transcriptional splicing in Figure 2A, plants RNAPII CTD is phosphorylated as transcription proceeds. However, in both humans and plants, unph RNAPII is recruited at the promoter region to form the PIC. After initiation, phosphorylation of RNAPII Ser5 CTD and Ser2 CTD begins as transcription proceeds toward the 3′ end. The RNAPII Ser5P CTD pauses at 5′SS, whereas RNAPII Ser2P CTD shows accumulation immediately after polyadenylation site (PAS), suggesting their role in splicing and transcription termination, respectively (Nojima et al., 2015; Zhu et al., 2018).

Despite similarities in the dynamics of RNAPII during transcription and co-transcriptional splicing among plants and humans, significant differences have also been reported, suggesting species-specific regulation of transcription and splicing (Hetzel et al., 2016). For instance, the engaged RNAPII profiles suggest, promoter-proximal pausing and divergent transcripts in Arabidopsis and maize are absent, whereas, these are prominent features of the human transcription (Core et al., 2008; Preker et al., 2008; Erhard et al., 2015; Zhu et al., 2015; Hetzel et al., 2016). In plants, the lack of promoter-proximal-pausing and a high correlation between transcription and steady-state RNA suggests initiation level regulation of transcription as compared to humans (Hetzel et al., 2016). In contrast to GRO-Seq analysis, the combination of GRO-Seq and pNET-Seq data in Arabidopsis show that RNAPII pauses or slows down in some genes after initiation of transcription (Zhu et al., 2018). However, unlike humans, which show RNAPII pausing in narrow regions (20–25 nt), plant RNAPII pausing in the promoter-proximal-regions is much broader (Figure 2C) (Zhu et al., 2018). Additionally, a strong positive correlation has been observed between RNAPII pausing at PAS, CpG methylation and longer genes in plants than in humans, which further suggests plant-specific regulation of transcription and splicing regulation (Hetzel et al., 2016).

Many features of transcription are conserved between humans and plants, however, some important differences exist between them. For example, there is a higher RNAPII elongation rate and AS in the presence of light than dark, demonstrating coupling between AS transcription and growth conditions, which is an important mechanism for plants to respond to different environmental conditions (Petrillo et al., 2014; Godoy Herz et al., 2019). Thus, the role of RNAP II processivity and its impact on AS needs to be analyzed in a tissue- and condition-dependent manner in plants. In the last decade, significant progress has been made to understand the co-transcriptional behavior of splicing/AS in animals, and yeast systems (Shukla and Oberdoerffer, 2012; Merkhofer et al., 2014; Saldi et al., 2016). However, this area is relatively new in plants and more studies are required to illuminate the co-transcriptional dynamics and its impact on RNA processing in tissue- and condition-specific manner.

## Aspects of IR and NMD in Plants and Humans

Intron retention is the most prevalent AS event in plants with observed frequencies between 28% to as high as 64% (Figure 1) depending upon growth condition, tissue type and the coverage of transcriptome data (Filichkin et al., 2010; Kalyna et al., 2012; Marquez et al., 2012; Mandadi and Scholthof, 2015). In comparison with plants, only 5% of IR events were observed in humans (Figure 1) (Keren et al., 2010; Reddy et al., 2012), owing to the large size of animal introns, sequencing depth and bioinformatics challenges to detect them. As a consequence, IR had received limited interest in humans until recently (Wong et al., 2013; Braunschweig et al., 2014; Boutz et al., 2015), whereas in plants IR has been found to be an important regulator in growth, development, physiology, and stress responses (Kalyna et al., 2012; Syed et al., 2012; Drechsel et al., 2013; Filichkin S.A. et al., 2015). However, recent research is unveiling various menace regulatory functions of IR in humans. For example, in addition to physiologically regulated events, any mutation in the splice site or splicing regulatory sequences cause aberrant IR, which further results in perturbed splicing patterns and potentially cause diseases (Jung et al., 2015; Wong et al., 2015; Jacob and Smith, 2017).

In humans, possible causes of IR and its abundance in response to cell differentiation and stresses have been studied recently (Wong et al., 2013; Braunschweig et al., 2014; Boutz et al., 2015). For instance, to predict the prevalence of IR, and their regulation and biological significance, a deep quantitative survey using Poly(A+) RNA-Seq data from 40 human and mouse tissue samples was conducted (Braunschweig et al., 2014). This study involved the quantitative measurement and comparison of reads across unspliced (exon–intron) and spliced (exon–exon) junctions, as well as, reads within introns in terms of “percent intron retention” (PIR) (Braunschweig et al., 2014). These findings suggest a large number of multiexonic genes are affected by the variable frequency of IR events processed in different tissues, which is much higher in comparison with previously estimated values (Pan et al., 2008; Wang et al., 2008). Comparative analysis across various species revealed tissue-specific IR events in neurons and immune cells. Furthermore, IR in neurons is highly conserved as compared with other AS events (Barbosa-Morais et al., 2012; Merkin et al., 2012; Braunschweig et al., 2014). In contrast with previous studies, IR was prevalent and mainly enriched in untranslated regions (UTRs), non-coding RNAs, depleted protein coding regions, and/or at the 3′ end of RNAs among different tissues in humans (Bicknell et al., 2012; Jacob and Smith, 2017). Moreover, the frequency of IR in the nucleus was observed to be higher than the cytoplasm, suggesting nuclear sequestration or coupling with the NMD pathway (Wong et al., 2013; Braunschweig et al., 2014; Boutz et al., 2015; Edwards et al., 2016).

In comparison with humans, the prevalence and significance of IR in plants and its role in development, stress and tissue-specific physiology are well documented. The observed frequency of IR in plants is as high as 64%, and potentially fine-tunes the transcriptome functionality (Filichkin et al., 2010, 2018; Kalyna et al., 2012; Drechsel et al., 2013; Filichkin S.A. et al., 2015). However, the mechanisms behind the high occurrence of IR in plants are still not very clear, yet many studies emphasize its significance in plants under normal, stress and various development and growth conditions. For example, the expression of *INTERMINATE DOMAIN 14* (*IDD 14*) isoforms controlled via IR mediate starch accumulation and utilization under cold stress in Arabidopsis (Seo et al., 2011). Similarly, cold-dependent IR in clock genes such as *CIRCADIAN CLOCK ASSOCIATED 1* (*CCA1*), *LATE ELONGATED HYPOCOTYL* (*LHY*) and *PSEUDO-RESPONSE REGULATOR7* (*PRR7*), modulate their transcript and protein abundance for *CCA1* (Seo et al., 2011; James et al., 2012). In wheat, the PECTIN METHYL ESTERASE INHIBITOR (PMEI), which secretes pectin for the cell wall, is also regulated by IR. Although, *PMEI* IR isoforms are found in almost all tissues but only anthers contained mature transcripts without IR, suggesting possible tissue-specific functionality of these transcripts (Rocchi et al., 2012). Similarly, studies in a *Marsilea vestita* (Boothby et al., 2013) and Arabidopsis (Filichkin and Mockler, 2012; Filichkin S.A. et al., 2015) provide a useful model to explain unproductive AS via IR. It has been demonstrated in *M. vestita* that some NMD insensitive IR transcripts remain in the nucleus as un-spliced mRNAs. Subsequently, these IR transcripts could be spliced and their translation results in a specific function, such as gamete development (Boothby et al., 2013).

Interestingly, many of IR PTC+ transcripts are not subjected to NMD in plants (Kalyna et al., 2012), suggesting regulatory functions. Components of the NMD machinery are highly conserved between plants and humans and its efficiency is strongly influenced by the pioneer round of translation (activity of ribosomes) (Shaul, 2015). However, it is intriguing that NMD responses are much less pronounced under stressful conditions in humans and plants, affecting the expression and translation of stress-responsive genes and splice variants (Trcek et al., 2013; Shaul, 2015). For example, inhibition of NMD mediates plant defense response during pathogen attack in Arabidopsis NMD mutants as they constitutively make more salicylic acid (SA) and show a heightened response after infection with *Pseudomonas syringae* (Rayson et al., 2012). However, mechanistic details of AS and its role via protein diversity in subverting a pathogen attack is not clear. Since the NMD pathway is translation dependent, slow engagement of different non-canonical transcripts with the ribosomal machinery may be the cause of their degradation. Intriguingly, in several model species including Arabidopsis, PTCs in the first and last intron appear earlier in their sequence than expected by chance alone, to keep the metabolic cost of producing truncated proteins and their subsequent degradation (Behringer and Hall, 2016). This data supports the notion that the appearance of earlier PTCs in introns seems to be favored by selection. Presence of PTCs in the first and last introns also points toward multiple features favoring degradation of non-sense transcripts (Behringer and Hall, 2016).

Interestingly, introns in plants UTRs also play a crucial role by affecting translation efficiency via a process called intron-mediated enhancement (IME). IME was proposed as a conserved phenomenon enhancing the translation efficiency of IR transcripts (Parra et al., 2011; Gallegos and Rose, 2015). For example, analysis of 5′ UTR introns identified an intron element in transcripts of the Mg^2+^/H^+^ ion exchange (MHX) gene in Arabidopsis, which further show an increase in translation efficiency (Akua and Shaul, 2013). In summary, differences in the frequencies of IR events suggest a varied mode of downstream processing and fates of IR transcripts in plants and humans. However, further work is needed to illuminate the mechanistic details of the IME mechanism.

## As and Protein Diversity in Humans: Supporting Evidence

Higher eukaryotes are diverse with varying degrees of biological complexity, nonetheless, the number of protein-coding genes is comparable between different species (Chen et al., 2014). Comparative sequencing and evolutionary studies between different eukaryotic species (including complex avian and mammals to species with fewer cell types) suggest a strong correlation between AS and organism complexity (Chen et al., 2014). AS plays a crucial role to enrich the expression of many genes and mediates various biological functions, pathways, and processes (Merkin et al., 2012; Weatheritt et al., 2012; Irimia et al., 2014). In humans, despite significant advancements in the field of transcriptome and proteome analysis techniques, the extent to which AS transcripts contribute to protein diversity remains unclear. However, renewed interest in humans has led to concerted efforts to illuminate this phenomenon in the recent past (Table 1). For example, isolation and sequencing of ribosome-bound transcripts have enabled researchers to delineate how the variety and abundance of mRNAs correlate with ribosomal recruitment (potentially translating mRNA). In a recent study, the ribosomal-engaged landscape of AS transcripts was surveyed using ribosomal-profiling in humans (Weatheritt et al., 2016). The ribosomal profiling data suggest transcripts with exon skipping events are present in medium to high abundance and thus likely to be translated. On the contrary, transcripts present in low abundance at the transcriptome level were not engaged with the ribosomes. This might be due to either the presence of introns in the low abundance transcripts, which remain in the nucleus (Braunschweig et al., 2014; Boutz et al., 2015) or incomplete RNA processing, preventing ribosomal engagement (Weatheritt et al., 2016). Similarly, other studies using Frac-Seq (subcellular fractionation and RNA-sequencing) (Sterne-Weiler et al., 2013) and TrIP-Seq (transcript isoforms in polysomes sequencing) (Floor and Doudna, 2016), also detected a large proportion of splice variants in the polyribosome fractions suggesting spliced isoforms play a significant role in controlling protein output in human cells. However, the degree to which ribosomal bound AS transcripts are translated and represented at the protein level is unclear. For example, pre-mRNA processing in the nucleus influences an isoform’s association with polyribosomes (Sterne-Weiler et al., 2013). Approximately 30% of mRNA processing events are differentially partitioned between cytoplasmic and polyribosome fractions (Sterne-Weiler et al., 2013). Moreover, differences in the polyribosome association are the result of a change in the *cis*-regulatory landscapes such as inclusion or exclusion of uORFs and Alu-elements in the 5′UTR, and microRNA target sites in the 3′UTR by AS (Sterne-Weiler et al., 2013). Similarly, TrIP-Seq analysis revealed that each transcript isoform harbors special regulatory features controlling ribosome occupancy and translation (Floor and Doudna, 2016). Floor and Doudna (2016) found robust translational control by 5′ UTRs between cell lines, whereas 3′ UTRs impact cell type-specific expression. This work also suggested that transcript isoform diversity must be considered when associating RNA and protein levels.

**Table 1:** Major studies deciphering the role of AS in protein diversity in humans and plants using different technique.

Study	Organism	Major technique used	Conclusion	References
Stochastic noise in splicing machinery	Humans	Computational analysis	Most AS is a consequence of stochastic noise in the splicing machinery, and has no functional significance	Melamud and Moult, 2009
Assessing the contribution of alternative splicing to proteome diversity	Plants	Computational analysis	AS contributes to transcriptome diversity but its contribution to protein diversity is limited	Severing et al., 2009
Isoform-specific recruitment to polyribosomes	Humans	Frac-Seq	Addition to translation AS plays role in sequestration and mRNA-decay	Sterne-Weiler et al., 2013
Assess the role of AS in proteome diversity	Humans	Computational analysis	Most genes have a single dominant isoform at the protein level, whereas homologous exons have important cellular roles	Abascal et al., 2015
Expression of protein coding gene isoforms at protein level	Humans	Computational analysis	Most highly expressed gene have single dominant isoform represented at the protein level	Ezkurdia et al., 2015
Ribosomal-engaged landscape of AS transcripts	Humans and mouse	Ribo-Seq	Majority of splice variants are translated into proteins	Weatheritt et al., 2016
Tunable protein synthesis by transcript isoforms	Humans	TrIP-Seq	Alternatively spliced isoform levels effects translation output	Floor and Doudna, 2016
AS mediated expansion of protein interaction capabilities	Humans	ORF-Seq and PPI	Large number of alternative isoforms in the human proteome are “functional alloforms”	Yang et al., 2016
Transcriptome survey and contribution of AS toward proteome diversity	Plants	RNA-Seq and Ribo-Seq	AS increases protein complexity, however, its contribution is lower in plants as compared to humans	Yu et al., 2016
Impact of AS on human proteome	Humans	RNA-Seq and SWATH-MS	IR reduces the protein diversity but fine-tunes the human proteome functionality	Liu et al., 2017
Relationship between AS and protein complexity	Humans	Computational analysis	Majority of alternatively spliced transcripts may not be translated into proteins	Tress et al., 2017a
AS contribution in transcriptome and proteome diversity in *Physcomitrella patens*	Plants	RNA-Seq and qRT-PCR	AS has a small effect on proteome diversity but shapes the transcriptome	Fesenko et al., 2017

Some proteomic studies contradict ribosome profiling data and argue that only a small fraction of splice variants are represented at the protein level (Abascal et al., 2015; Ezkurdia et al., 2015; Tress et al., 2017a). Furthermore, the shotgun MS techniques used in many proteomic studies have their own limitations of coverage and sensitivity to detect low abundance splice variants at the protein level (Bensimon et al., 2012; Rost et al., 2015). To improve isoforms detection efficiency, alternative approaches need to be developed to overcome the limitations of the techniques used at present. Toward this goal, full-length ORFs of AS isoforms from a large number of human genes were cloned and protein–protein interaction (PPI) profiling was performed to demonstrate the functionality of hundreds of protein isoforms (Yang et al., 2016). This study demonstrated vastly different interaction profiles among isoforms as a result of AS. Strikingly, the isoforms encoded by the same genes exhibit widespread functional differences in the PPI network analysis. Since differences between protein isoforms are as high as observed between different genes, isoforms-specific partners could have different expression and functional characteristics. Yang et al. (2016) proposed that a vast diversity of “functional alloforms” are generated that contribute to different physiological and developmental processes (Yang et al., 2016).

In humans, a number of studies have been conducted to identify protein isoforms that result from AS by comparing transcriptome and proteome data (Brosch et al., 2011; Ezkurdia et al., 2012; Lopez-Casado et al., 2012; Sheynkman et al., 2013). However, most of these studies were carried out in a steady state manner and do not explain the consequences of perturbation in splicing to protein diversity. To overcome these limitations, an integrated approach was developed to illuminate how variation in mRNA splicing patterns could subsequently change the proteome composition in a systematic manner (Liu et al., 2017). Selectively depleted spliceosome U5 component PRPF8 (Wickramasinghe et al., 2015) orchestrated changes at the transcriptome and proteome level that were determined using RNA-Seq and Sequential Window Acquisition of all Theoretical Spectra-Mass Spectrometry (SWATH-MS), respectively. After PRPF8 depletion, quantification of splice variants and a large fraction of proteome identified 1,542 proteins that displayed at least one peptide with altered expression. Functional annotation revealed that transcripts with altered splicing patterns possess similar cellular functions and processes (such as RNA splicing, the mitotic cell cycle and ubiquitination) as those found in proteins with altered levels. Thus, splicing variants at the transcriptomic level were found to be functionally represented at the protein level (Liu et al., 2017). Furthermore, to identify the differentially spliced event at the transcriptome level, the authors used a transcript-centric approach, in which a transcript is considered as a whole unit (Liu et al., 2017). Firstly, transcript expression is estimated, followed by identification of differentially used transcripts and expressed genes. The correlation analysis between fold changes in the expression level after PRPF8 depletion suggests protein expression levels are exclusively associated with the alternatively spliced transcripts involving differential transcripts usage (DTU). Interestingly, IR events, which are considered as one of the major regulatory events for gene expression, had reduced representation at the protein level (Liu et al., 2017). Although, around 75% of multi-exon genes are affected by IR and help in regulating transcript levels (Braunschweig et al., 2014), its impact on protein expression is inverse because an increase in the level of IR transcripts, throughout the genome, is associated with PRPF8 depletion (Wickramasinghe et al., 2015). The peptide expression of 270 genes with retained introns showed downregulation of protein expression coded by genes with IR. Moreover, the relative abundance of transcripts also plays a significant role in protein expression as the low abundance transcripts with IR do not affect the protein expression until they are present in high abundance. These observations suggest IR reduces the protein diversity but fine-tunes the human proteome functionality. However, this finding may not be strictly applicable to plants as IR is the predominant mode of AS and may fine-tune the proteome function via modulating its abundance, especially in stressful conditions.

Collectively, various studies in the recent past such as ribosomal profiling (Weatheritt et al., 2016), PPI interaction analysis of spliced isoforms (Yang et al., 2016), and integrative analysis using perturbed systems (Liu et al., 2017) suggest a strong correlation between AS and protein diversity in humans. Moreover, these studies provide an alternative to MS techniques, which have limitations of coverage and sensitivity to detect low level splice isoforms at the protein level and could be useful to study plant systems in the future.

## As and Protein Diversity in Humans: Opposing Evidence

The contribution of AS toward protein diversity in humans is well documented (Weatheritt et al., 2016; Yang et al., 2016; Blencowe, 2017; Liu et al., 2017). However, recent data from some proteomic studies in humans supports the opposing view and suggest that AS may not be the key contributor to protein diversity (Tress et al., 2017a,b). Substantial amount of AS data has been generated in various RNA-Seq experiments in humans, however, most of the alternative isoforms in proteomic experiments are undetectable even in large-scale MS-based analyses (Ezkurdia et al., 2015; Tress et al., 2017a,b). Moreover, some studies suggest that AS is the result of noise in the splicing machinery and does not contribute to protein diversity as expected. For example, Melamud and Moult (2009) proposed a stochastic noise model of splicing machinery, which explained that AS events arise as a result of noise in the splicing machinery (Melamud and Moult, 2009). The idea of noise in the splicing machinery has also been supported by other studies as well, suggesting a large proportion of alternative isoforms are non-functional (Modrek et al., 2001; Kan et al., 2002; Neverov et al., 2005). Further, it was recently demonstrated that the majority of expressed genes have a single major isoform represented at the protein level (Abascal et al., 2015; Ezkurdia et al., 2015). This was supported by monitoring peptide evidence from eight large-scale MS experiments and observing that only one main protein isoform was dominant at the protein level from almost all coding genes (Tress et al., 2017a). On the other hand, several reports have supported the presence of a small number of alternative protein isoforms in humans (Tanner et al., 2007), drosophila (Tress et al., 2008), and mouse (Brosch et al., 2011) in large-scale proteomic studies. However, AS events such as ES detected in RNA-Seq studies have revealed subtle effects on the structure and function of proteins. Tress et al. argue that it is the gene expression that is conserved across species, have strong tissue dependence, and are translated to detectable proteins but not the alternatively spliced isoforms (Tress et al., 2017a,b). Clearly, more work and evidence is needed to illuminate the relationship between AS and protein diversity in tissue- and condition-dependent manner.

The efficiency of the MS also needs to be enhanced because current MS techniques cannot reliably detect changes in protein domains as a result of AS (Wang et al., 2018; Chaudhary et al., 2019). For example, lysine and arginine coding triplets are the most abundant amino acids at the end of exons or exon–exon junctions (Wang et al., 2018), and are the preferential sites for trypsin, which is the most common enzyme used in MS analyses (Olsen et al., 2004). Since trypsin digests exon–exon junctions, it hinders with the detection of novel AS derived peptides in MS-based proteome analysis (Ning and Nesvizhskii, 2010; Sheynkman et al., 2013; Wang et al., 2013). To improve efficiency, enzymes such as chymotrypsin can be used as an alternative to improve the detection of AS-derived peptides in proteome studies (Wang et al., 2018; Chaudhary et al., 2019).

## The Contribution of as Toward Protein Diversity in Plants

The role of AS in the expansion of functional protein diversity is less clear in plants as compared to humans (Kim et al., 2007). However, in the absence of in-depth proteomic studies to elucidate the role of AS toward protein diversity is tenuous. Recently, some studies have evaluated the influence of AS on protein diversity in plants. For example, hypoxia in Arabidopsis mediates an increase in the number of IR events in many mRNA isoforms, and show ribosomal engagement and potentially influence protein variety and abundance (Juntawong et al., 2014). Interestingly, transcriptome and translatome profiling among shoot apical meristem (SAM) and leaf domains, suggest 751 genes isoforms show domain-specific enrichment in the translatome data (Tian et al., 2019). Another study in Arabidopsis has shown that 35% of AS events are represented among the polysome-bound mRNAs and expected to undergo translation (Yu et al., 2016). Among all transcripts, IR is the least representative among translated transcripts, compared with untranslated transcripts, suggesting a variable role of IR in regulating transcript level via NMD machinery or sequestration in the nucleus and further processing on demand (Filichkin S.A. et al., 2015; Filichkin S. et al., 2015). In contrast, other splicing events such as ES, 5′AD, and 3′AA have higher proportions among transcripts that may be translated (Yu et al., 2016). Sequence analysis of translated transcripts suggests that any alteration in the CDS by AS could lead to a change in protein sequences (Yu et al., 2016). Interestingly, a large proportion of a new class of exon-like introns called exitrons (Marquez et al., 2015) (Figure 1) was found at the transcriptome as well as translatome level, suggesting these unique events of AS may contribute to protein diversity (Yu et al., 2016). A recent report in *Physcomitrella patens* suggests that AS shapes the transcriptome rather than the proteome (Fesenko et al., 2017), because only 85 isoform-specific peptides, representing only 25 differentially AS genes, were found in moss cells. Among all, only five genes unambiguously showed two or more protein isoforms from the same locus. The number of AS genes identified in this study was substantially large (approximately 66 times) as compared to proteomic datasets, nonetheless, only support a small contribution of AS on protein diversity. Collectively, these data support the view that AS increases protein complexity, however, its contribution is found to be lower as compared with humans (Yu et al., 2016). Further, supporting as well as the opposing evidence presented above for the notion, “AS contributes toward protein diversity,” suggests that the exact number of splice isoforms represented at the proteome level in humans as well as in plants is still elusive. On the other hand, IR events are the predominant AS type in plants and may not be translated due to nuclear sequestration or degradation by the NMD pathway and thus remain poorly represented in MS experiments (Gohring et al., 2014; Hartmann et al., 2018). Since limited information is available at the proteome level, we envisage that strategies like cloning of spliced isoforms and PPI profiling (like in humans Yang et al., 2016), could be beneficial and may uncover different aspects of AS contribution toward protein diversity in plants.

## Splicing Memory and Plant Stress Tolerance

**FIGURE 3:**
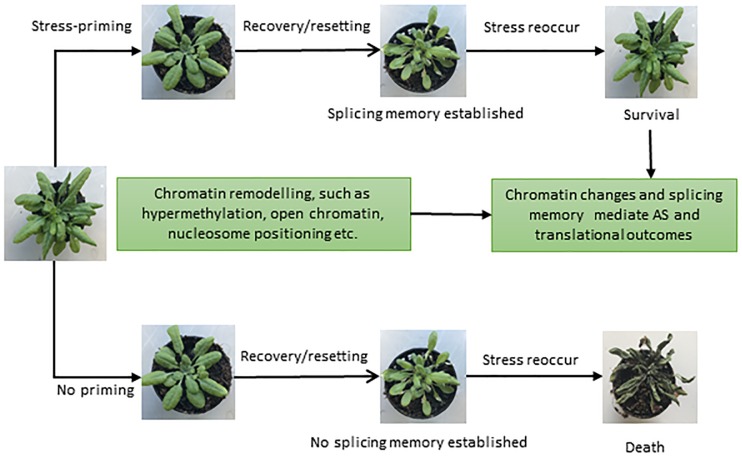
Different phenotypes representing the importance of splicing memory in plants. Once exposed to stressful conditions, plants develop an adaptive componenet of induced resistance defined as stress-priming. Stress-induced chromatin modifications plays a crucial role in stress-priming and likely help in establishing a splicing memory, which in turn facilitates plant survival upon exposure to recurring stresses (upper panel). In the absence of priming (lower panel) and splicing memory, plants may die once the stress reoccur. Different phenotypes shown are based on Ling et al. (2018) and Sanyal et al. (2018).

Successful attempts have been made in plant systems to understand the impact of stress, its tolerance and the development of genetically engineered stress tolerant crops (Vinocur and Altman, 2005; Pereira, 2016). However, the majority of studies are restricted to acute and single stress only (Zhu, 2016). Since stresses are usually multiple, recurring and chronic, plants have evolved sophisticated defense mechanisms to deal with a variety of stresses. Plants have the ability to acquire tolerance to chronic stress through establishing “molecular stress memory” to confer tolerance through a phenomenon referred to as priming or acclimation, in response to previous exposure to a mild stress (Sani et al., 2013; Conrath et al., 2015; Hilker et al., 2016). Priming establishes a new cellular state in plants, which is different from the naïve or unexposed plants (Sani et al., 2013; Conrath et al., 2015; Hilker et al., 2016). In recent years, it has become increasingly apparent that various epigenetic features, such as chromatin modifications, nucleosome positioning, and DNA methylation, are important components of adaptation and play a role in stress memory (Boyko et al., 2010; Ding et al., 2012; Lämke and Bäurle, 2017; Friedrich et al., 2018). Since the splicing process is largely co-transcriptional in nature, the chromatin structure has a strong influence on the transcriptional as well as the splicing processes (Listerman et al., 2006; Khodor et al., 2011; Jabre et al., 2019). Recent DNase I-Seq data suggest enrichment of IR in DNase I hypersensitivity sites (DHSs) in both Arabidopsis and rice (Ullah et al., 2018). Since RNAPII elongation speed is high in regions with open chromatin, the spliceosome machinery has less time to recognize introns, resulting in more IR during co-transcriptional splicing (Braunschweig et al., 2014; Naftelberg et al., 2015). Furthermore, condition-dependent variation in the chromatin environment under different stresses and environmental cues plays an additional regulatory and fine-tuning role (Struhl and Segal, 2013; Zentner and Henikoff, 2013). Moreover, along with the positioning and spacing of the nucleosome, posttranslational modifications and DNA methylation also affect the transcriptional and splicing dynamics (Naftelberg et al., 2015; Friedrich et al., 2018; Zhang et al., 2018). Hence, various epigenetic modifications may provide a basic regulatory mechanism to orchestrate stress and splicing memory (Figure 3) in the same or future generations to respond to recurring stress more efficiently.

Not surprisingly, a recent study uncovered splicing memory response to heat stress priming in Arabidopsis as revealed by genome-wide differentially expressed genes (DEGs) and AS patterns (Ling et al., 2018; Sanyal et al., 2018). DEGs in response to heat stress were identified for different stages of priming, and genes responsible for potentially controlling heat stress memory were selected. With the help of gene networking analysis, heat and abiotic responsive genes were found to be involved in stress memory (Ling et al., 2018). Importantly, IR was found to be the most prevalent event under heat stress and contributed significantly toward establishing the splicing memory in response to heat. The primed plants produced comparable splicing patterns and efficiency compared with control plants, which were not exposed to heat stress before. In contrast, non-primed plants showed a significant increase in IR and produced splicing variants in heat conditions. Therefore, the primed plants, after relief from the second exposure to heat stress, maintain the splicing memory and perform in a similar manner to the control plants under non-stressful conditions (Ling et al., 2018). Ling et al. (2018) suggested that heat stress priming might be established at the post-transcriptional level and maintains splicing memory, which is crucial for plant survival and adaptation under stress. It is tempting to speculate that exposure to multiple stresses and coordination of gene expression and splicing patterns mediated by the chromatin environment may influence predictable responses and adaptive solutions in the long term. However, further research is needed to explore splicing memory and the underlying molecular mechanisms in response to different stresses in plants. We envisage that in addition to its contribution to protein diversity, AS may also play regulatory roles, and after repeated episodes of stress, splicing memory may also fine-tune stress-specific protein diversity to enhance plants networking capability to cope with given stress.

## Conclusion

Emerging evidence indicates that the splicing process is also predominantly co-transcriptional in plants as in humans (Zhu et al., 2018). In plants, environmental fluctuations modulate chromatin structure, which in turn, could influence the co-transcriptional splicing process. Intriguingly, recent work indicates that plants can establish splicing memory in response to higher temperature conditions and thus may “remember” a particular stress, likely through specific epigenetic signatures. This strategy may allow plants to engender an appropriate and reproducible response to a given stress. Further, IR transcripts are prevalent in plants and a majority of these are “trapped” in the nucleus. In addition, IR and many other AS transcripts are NMD sensitive and potentially degraded by the NMD pathway. It is clear that AS modulates transcriptome composition and splicing ratios, however, its role in diversifying proteome complexity is far from being understood.

It was a surprising discovery to find that the human genome codes for only ∼20,000 to 21,000 protein-coding genes (Willyard, 2018), which is comparable with a weed (Arabidopsis, which has over 27,000 protein-coding genes) with a much smaller genome (Swarbreck et al., 2008). Since 95% of human genes and over 70% of genes in some plants are alternatively spliced, they can potentially make multiple proteins from each gene and considerably increase their proteome complexity (Kim et al., 2007; Pan et al., 2008). Although it is clear that AS does increase proteome complexity, the extent to which it enhances proteome diversity is far from clear. Multiple proteomic studies do not support a linear relationship between splicing and proteome complexity in humans (Tress et al., 2017a,b). Therefore, in-depth proteome analyses in multiple tissues and conditions, in conjunction with the variable expression of corresponding genes, need to be performed to illuminate the relationship between AS and proteome complexity in plants.

## Author Contributions

All authors listed have made a substantial, direct and intellectual contribution to the work, and approved it for publication.

## Conflict of Interest Statement

The authors declare that the research was conducted in the absence of any commercial or financial relationships that could be construed as a potential conflict of interest.
